# Supplementary figures and images for: Probing T-cell response by sequence-based probabilistic modeling
Source: PLoS Comput Biol. 2021 Sep 2;17(9):e1009297. doi: 10.1371/journal.pcbi.1009297 (PMC8476001; doi:10.1371/journal.pcbi.1009297)

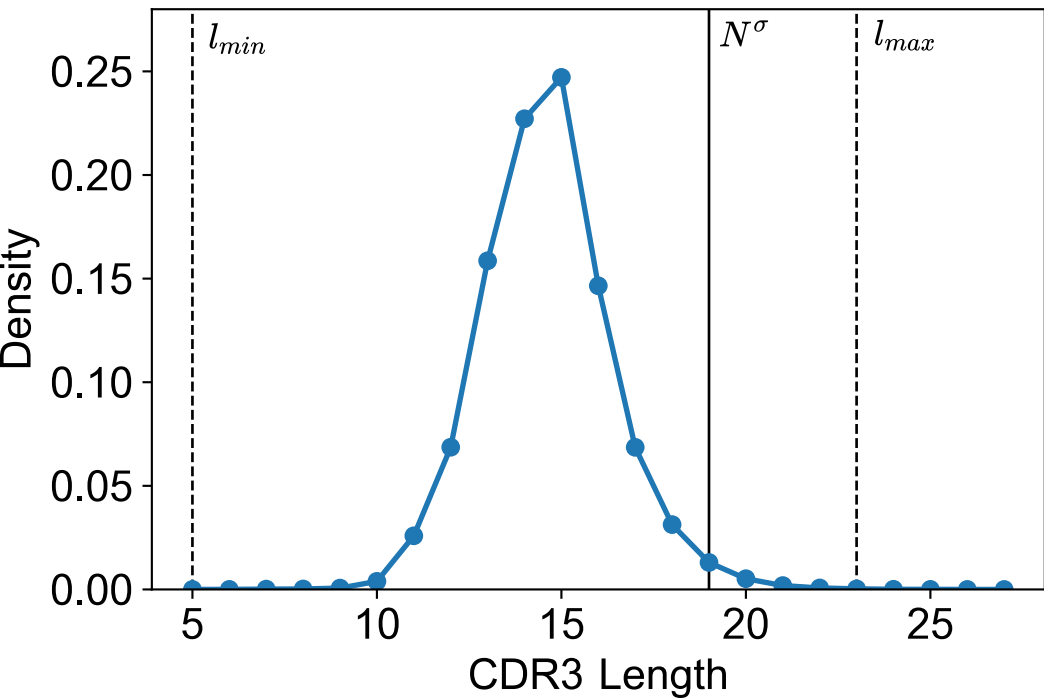

Supplement: S1 Fig — CDR3 length distribution in the full dataset of 276993 sequences from Ref. [16]. (PDF) [file pcbi.1009297.s001.pdf]

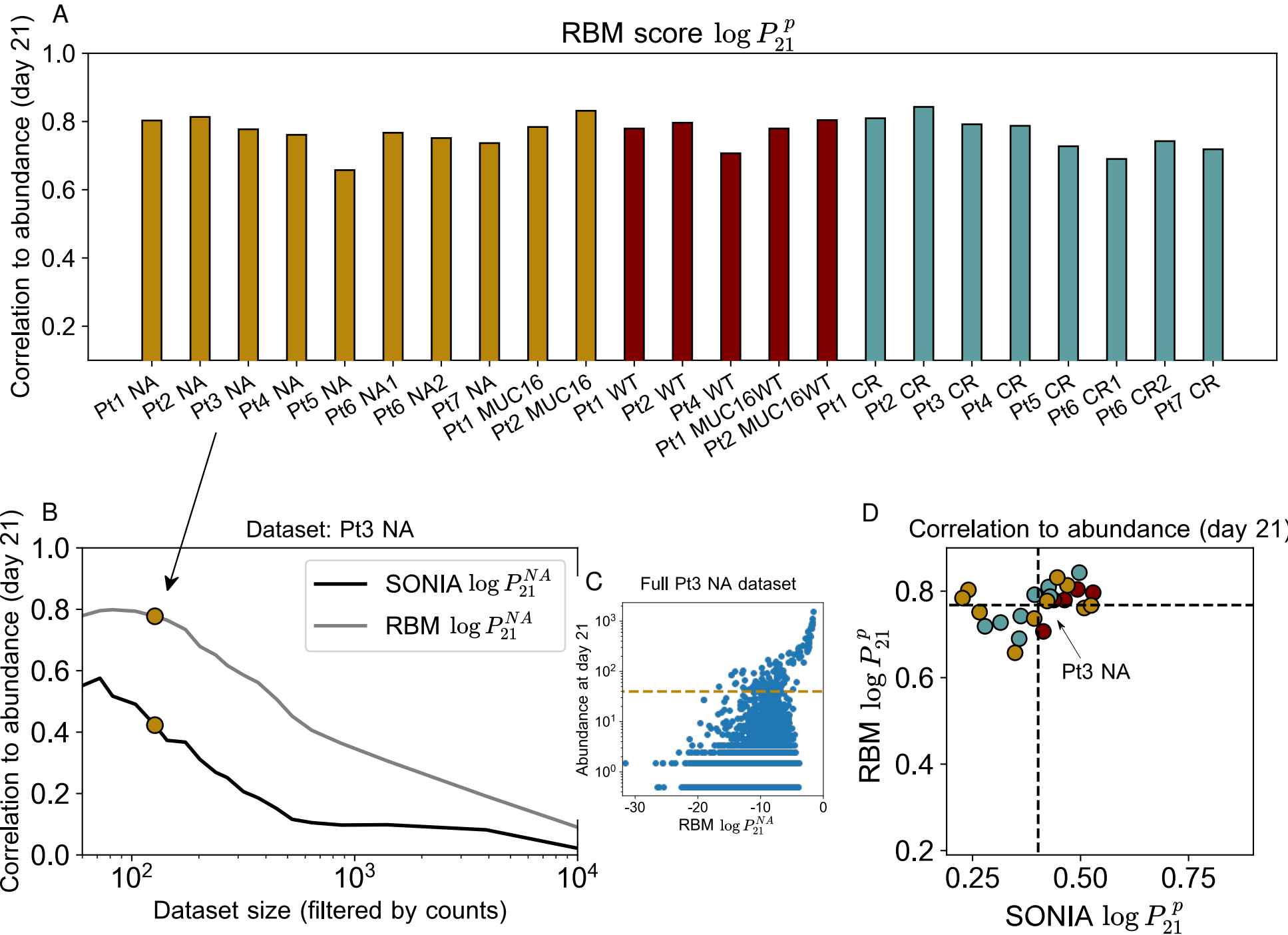

Supplement: S3 Fig — A: Correlation coefficient of the RBM likelihood logP21p with clonal abundances at day 21 post-stimulation in all samples from [16]. B: The correlation coefficient shown is obtained by progressively filtering out the low-abundance sequences from the testing set, as illustrated for the Pt3 NA dataset. C: Scatter plot between clone abundance at day 21 and the RBM likelihood logP21NA for the full Pt3 NA dataset, showing that the correlation is poor for clones at low counts. The golden dashed line marks the minimal clone abundance that is considered to measure the correlation coefficient reported in A and indicated by the golden dot in B. B-D contain the comparison between the performance of the RBM and SONIA, confirming that capturing sequence correlations (as the RBM does, see also S4 Fig) ensures better prediction of clonal abundance. (PDF) [file pcbi.1009297.s003.pdf]

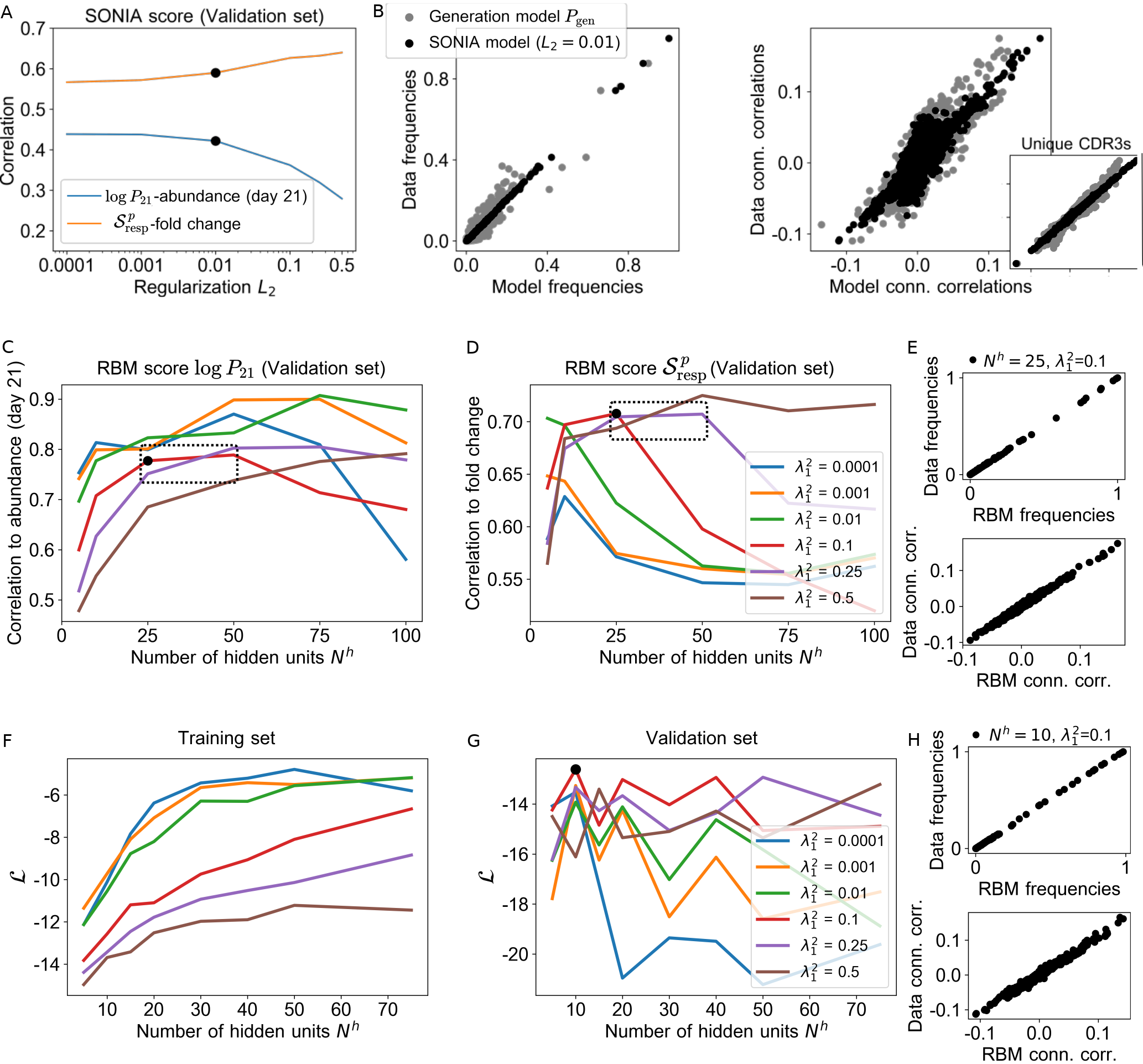

Supplement: S4 Fig — A: Hyperparametric search for training SONIA on count-weighted datasets: we set the L2 regularization at the intermediate value 0.01 (black dot), which ensures good performance at capturing both clone abundance (through logP21p) and clonal fold change (through Srespp) in the validation set. B. Data statistics (single-site frequency and connected correlations) are reproduced by SONIA. SONIA is documented to perform best when trained on lists of unique sequences [15], where also correlations are well reproduced (see inset). As a comparison, the gray points stand for the marginals given by the generation model Pgen. C-D: Hyperparametric search for training RBM on count-weighted datasets. Similarly to A, it is performed based on how clonal abundances and clone fold change are reproduced by logP21p and Srespp. We identify as optimal the combinations of parameters indicated by the black box, which result in maximal correlations with fold change (D) while keeping the correlation to abundance in the intermediate-high range (C). F-G: Hyperparametric search for training RBM on datasets of only responding CDR3s. Optimal Nh and λ12 (black dot) are chosen to ensure high likelihood L on the training set and to prevent overfitting on the validation set. E-H show that in both cases single-site amino acid frequencies and connected correlations are extremely well reproduced by the RBM (with parameters marked by the black dot in D and G). In all hyperparametric searches shown, the model training set consist of 80% of all sequences in the dataset, the validation set is the remaining 20%. Datasets used: Pt3 NA from [16] (A-E), samples from Ref. [17] (F-H). (PNG) [file pcbi.1009297.s004.png]

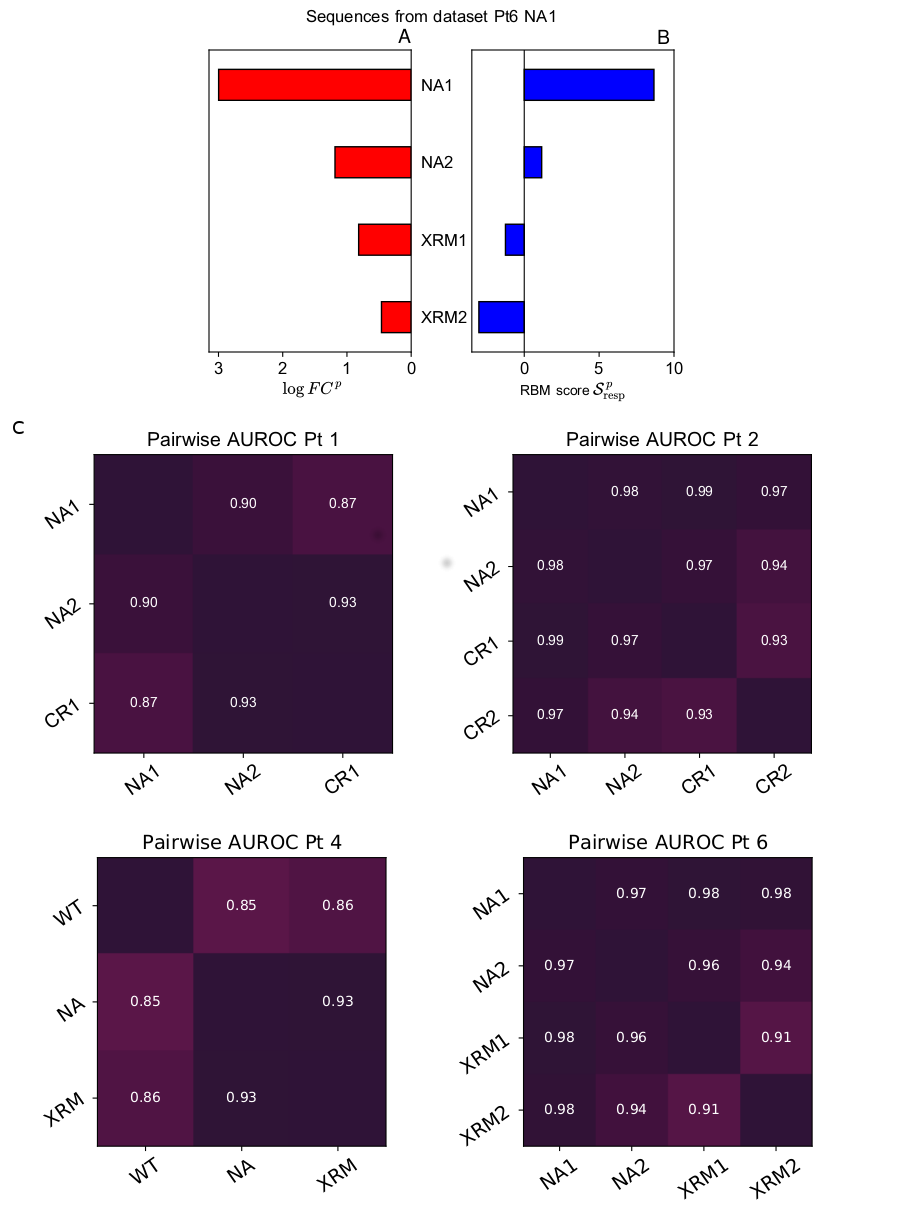

Supplement: S5 Fig — A-B: Differential degree of clone expansion under stimulation p is mapped by the model into differential response scores Srespp, enabling a model-based assessment of response specificity. Here we consider sequences from sample Pt6 NA1, where clone abundance reflects response to NA1. The fold change due to NA1 stimulation, FCNA1, is on average higher than the fold change measured for all the other antigens p tested in the same patient, FCp (A), suggesting a specific response. This set of NA1-specific responders is on average assigned higher response score by the RBM model trained on the Pt6 NA1 dataset, SrespNA1, than by models trained on the other Pt6 samples, Srespp, where the same sequences behave as unspecific responders (B). All models are trained on 80% of a given sample, and we show average log fold change log FCp and response scores Srespp (both expressed as log base 10) over the Pt6 NA1 testing set. C: Matrices of RBM pairwise AUROC for Pt1, Pt2, Pt4 and Pt6 (patients for whom more than 2 antigens were tested, see Table 1). The value at each row/column intersection gives the AUROC (estimated through RBM scores) of response specificity between the antigen to which the row and column refer. (PNG) [file pcbi.1009297.s005.png]

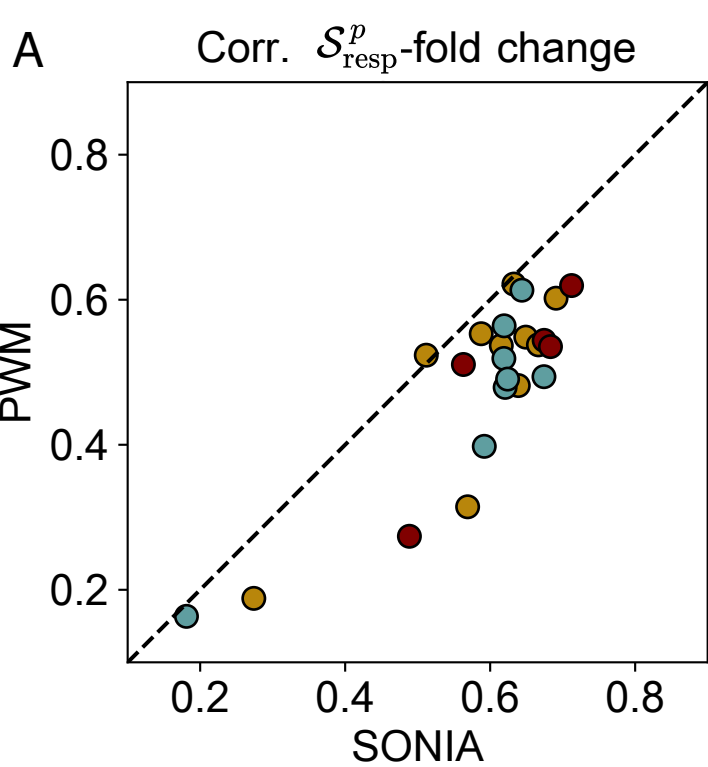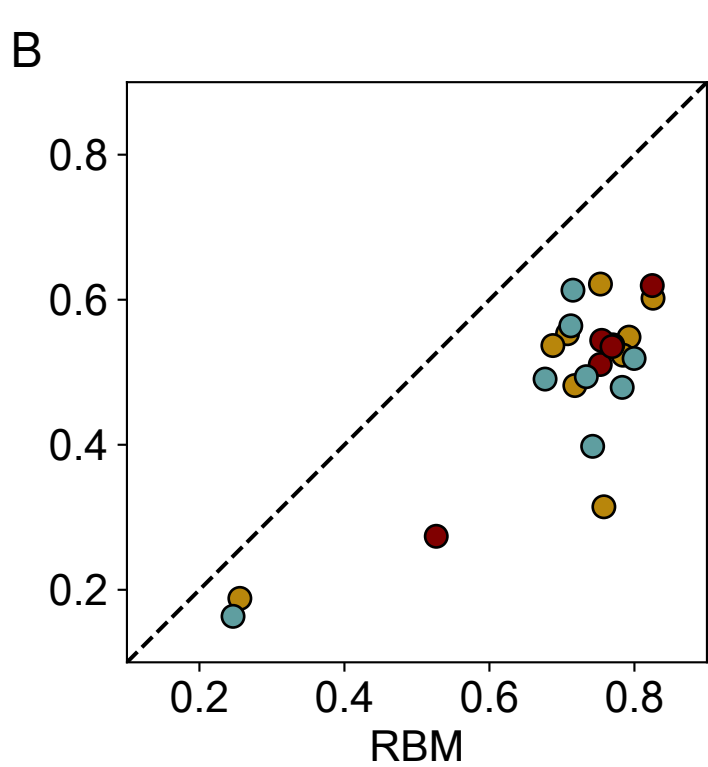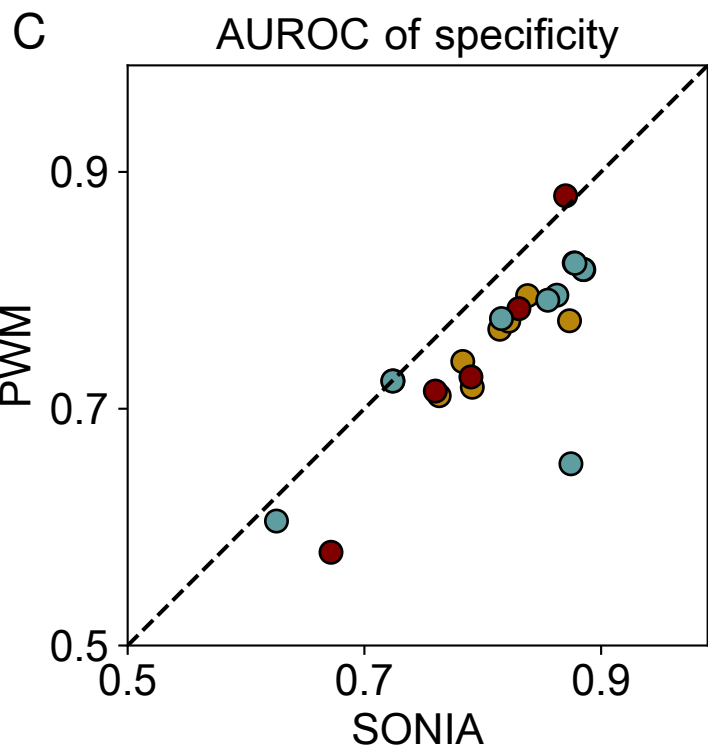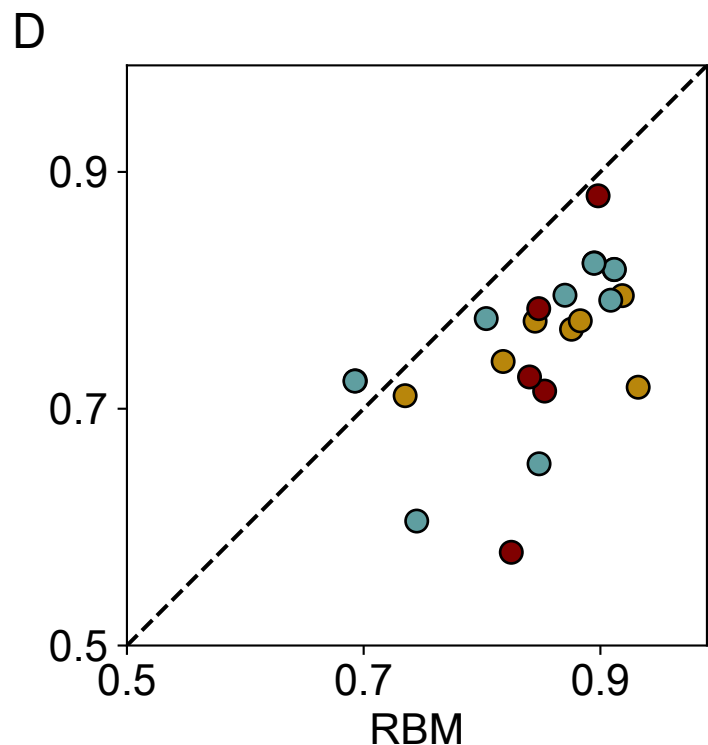

Supplement: S6 Fig — PWM is the simplest sequence-based modeling strategy and it is here learnt on aligned count-weighted datasets. The PWM probability assigned to each CDR3 clone σ is factorized over CDR3 positions, i.e. Ptp(σ)=∏i=1NσPt,ip(σi) and Pt,ip(σi) is taken as the frequency of amino acid σi at position i in the p-specific repertoire at time t = 0, 21 (days). PWM performance in terms of correlation between response score Srespp and clone fold change (see Fig 3) and AUROC of specificity (see Fig 4) is compared to SONIA (A,C) and RBM (B,D) for all samples from [16]. (PDF) [file pcbi.1009297.s006.pdf]

# Tetramer-sorted TRB data

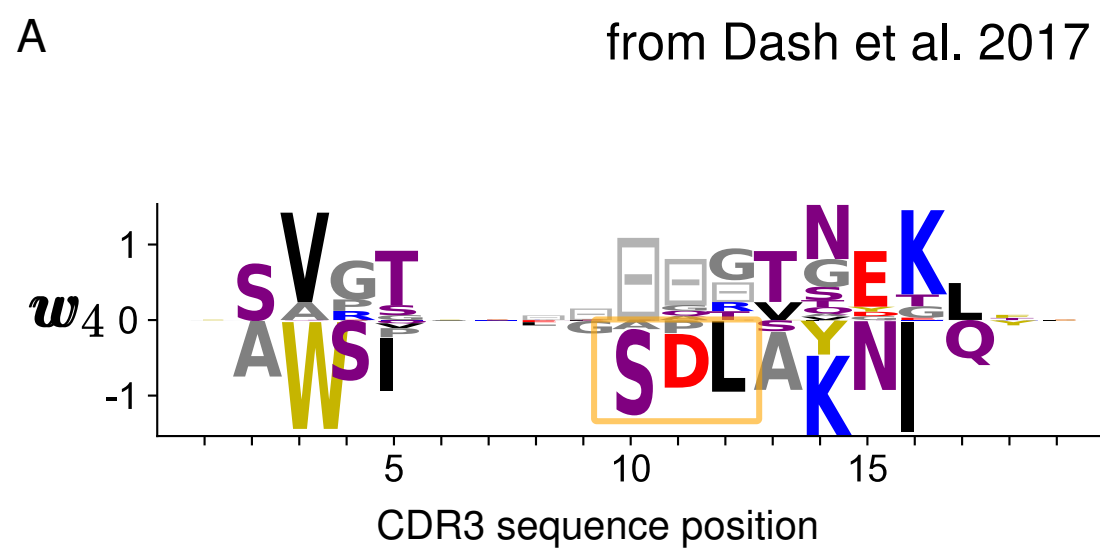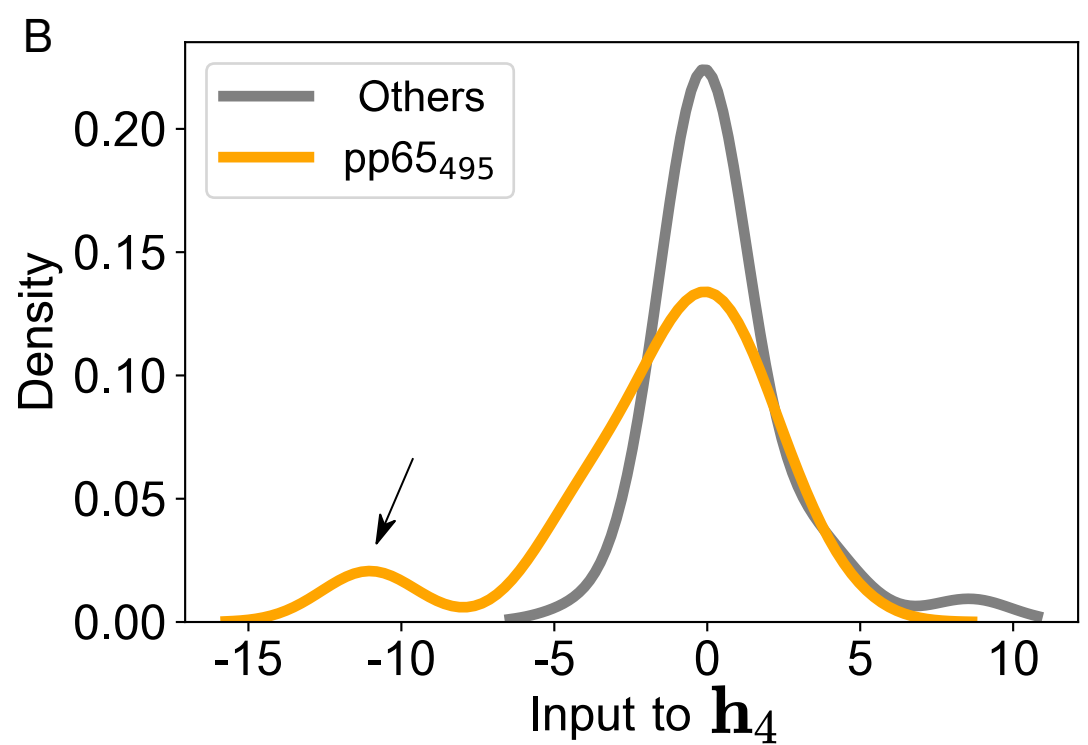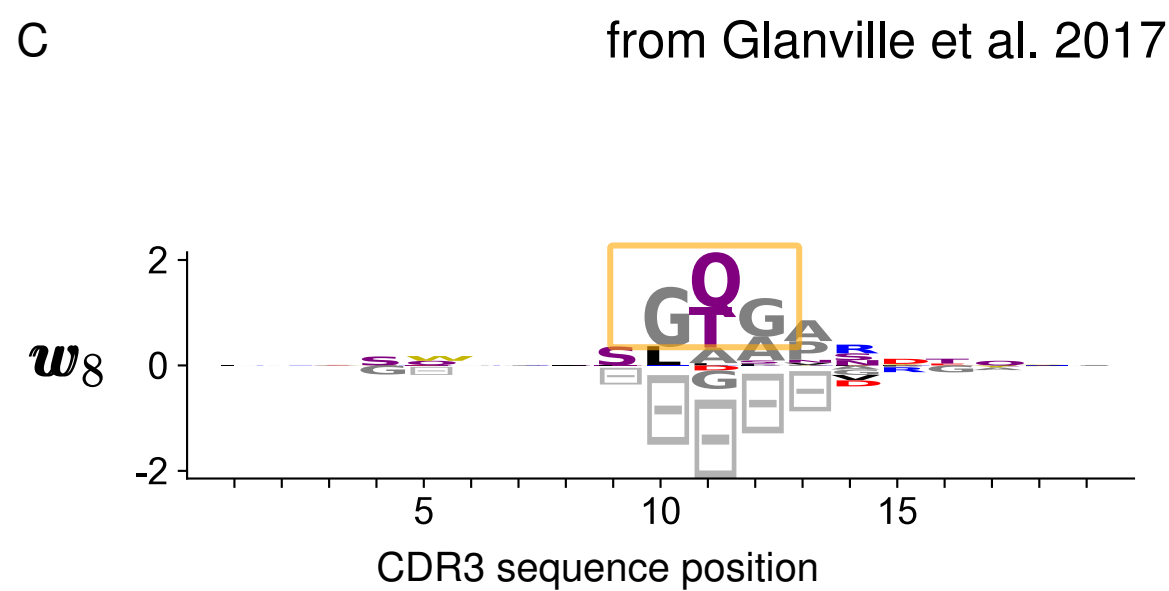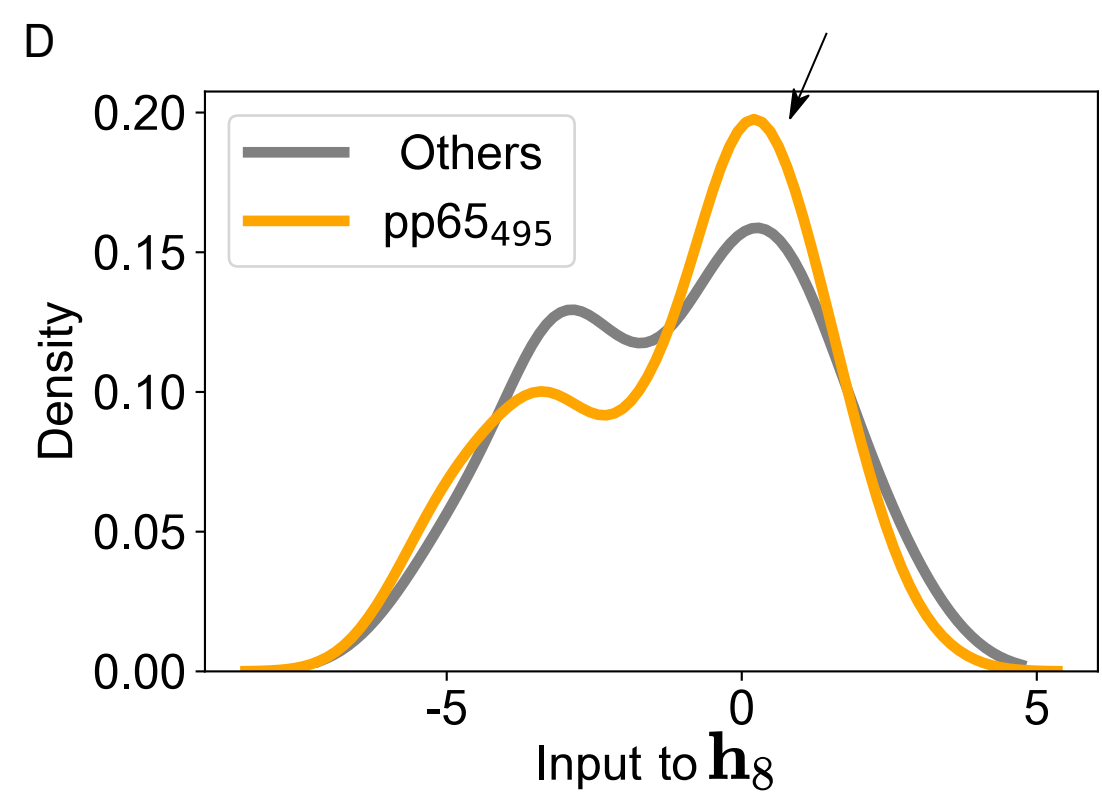

## *M. tuberculosis*-specific TRB data from Glanville et al. 2017

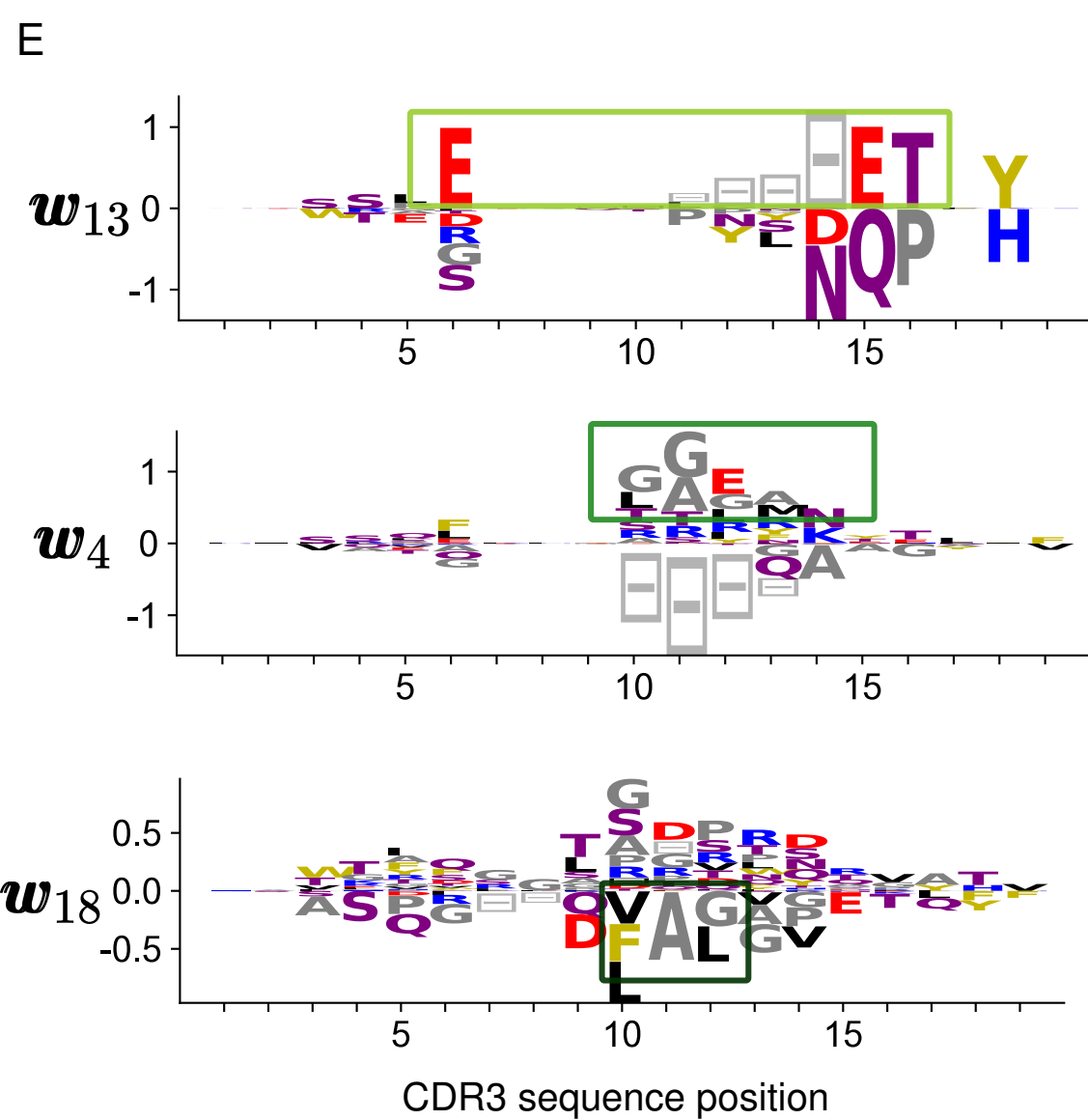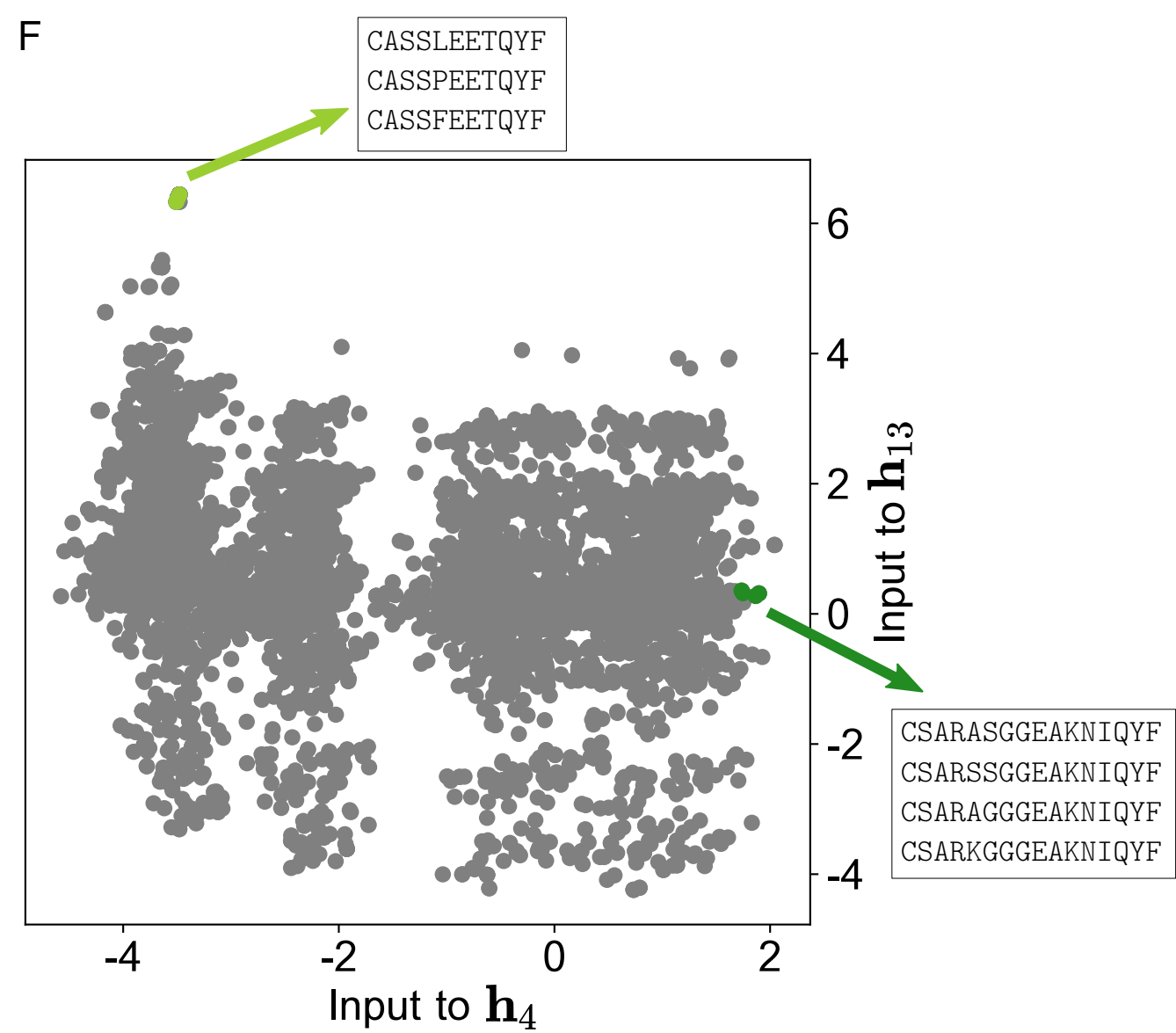

Supplement: S7 Fig — A: One of the set of weights (w4) learned by the RBM (same model as in Fig 6B) picks up the pp65495 motif discussed in [17]. B: The clones characterized by this motif can be identified by the negative values of the projection onto this weight (input to h4), indicated by the black arrow. C-D: Same representation as in A-B for the RBM trained on EBV, CMV, Flu data from [22] (same model as in Fig 6D), where one weight highlights the sequence motif found in [22] for the pp65-specific clones. E: Sets of weights of a RBM trained on the CDR3 sequences of M. tuberculosis-specific CD4+ T cells from [22] recover three motifs from the five representative TCR specificity groups analyzed in [22]. The set of weights w4 picks up a gap motif (symbols ) indicating that the motif ‘GGE’ is found in the center of longer CDR3 sequences, where there are gap insertions in the other sequences. F: We choose for illustration two specificity groups: their sequences (listed in the boxes) are characterized by high values of the projection onto the corresponding weights (here the inputs to h4 and h13). (PDF) [file pcbi.1009297.s007.pdf]

TRB response to NA (Day 21, Pt3)

TRB response to CR (Day 21, Pt5)

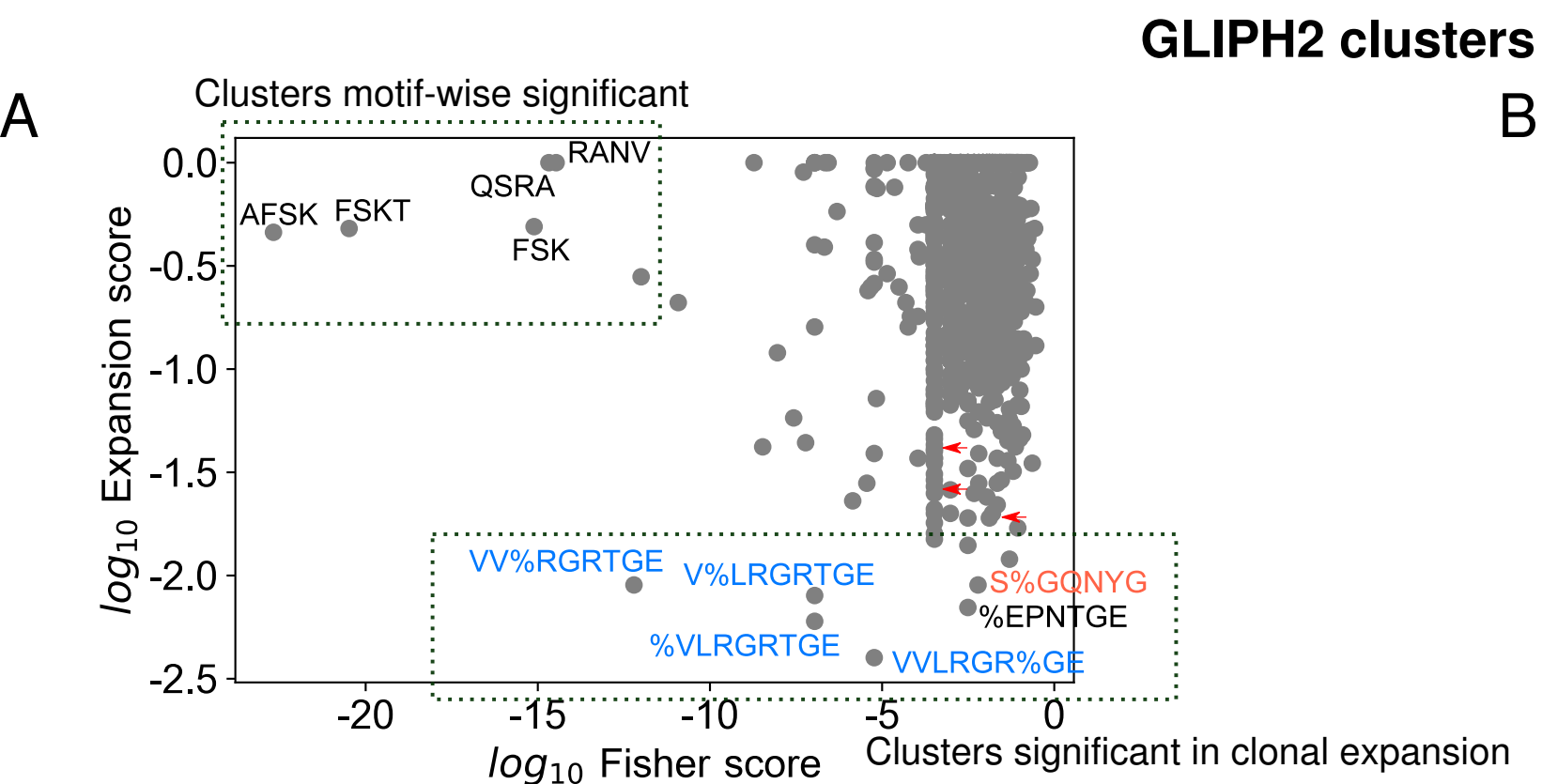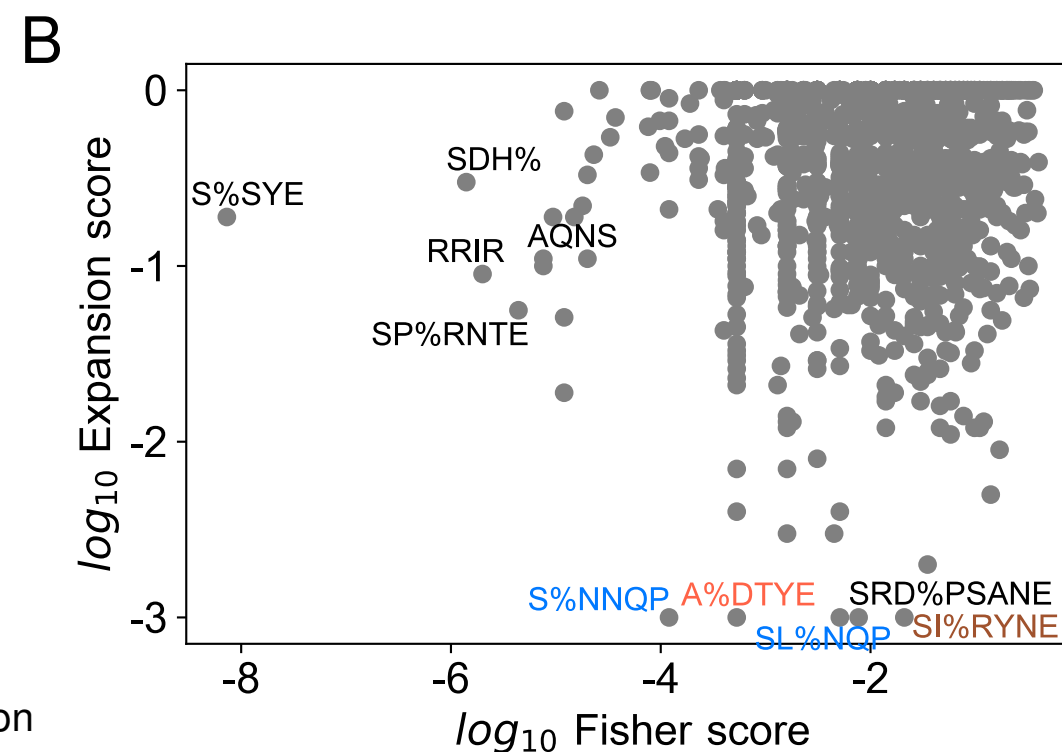

**C**

RBM dimensionality reduction

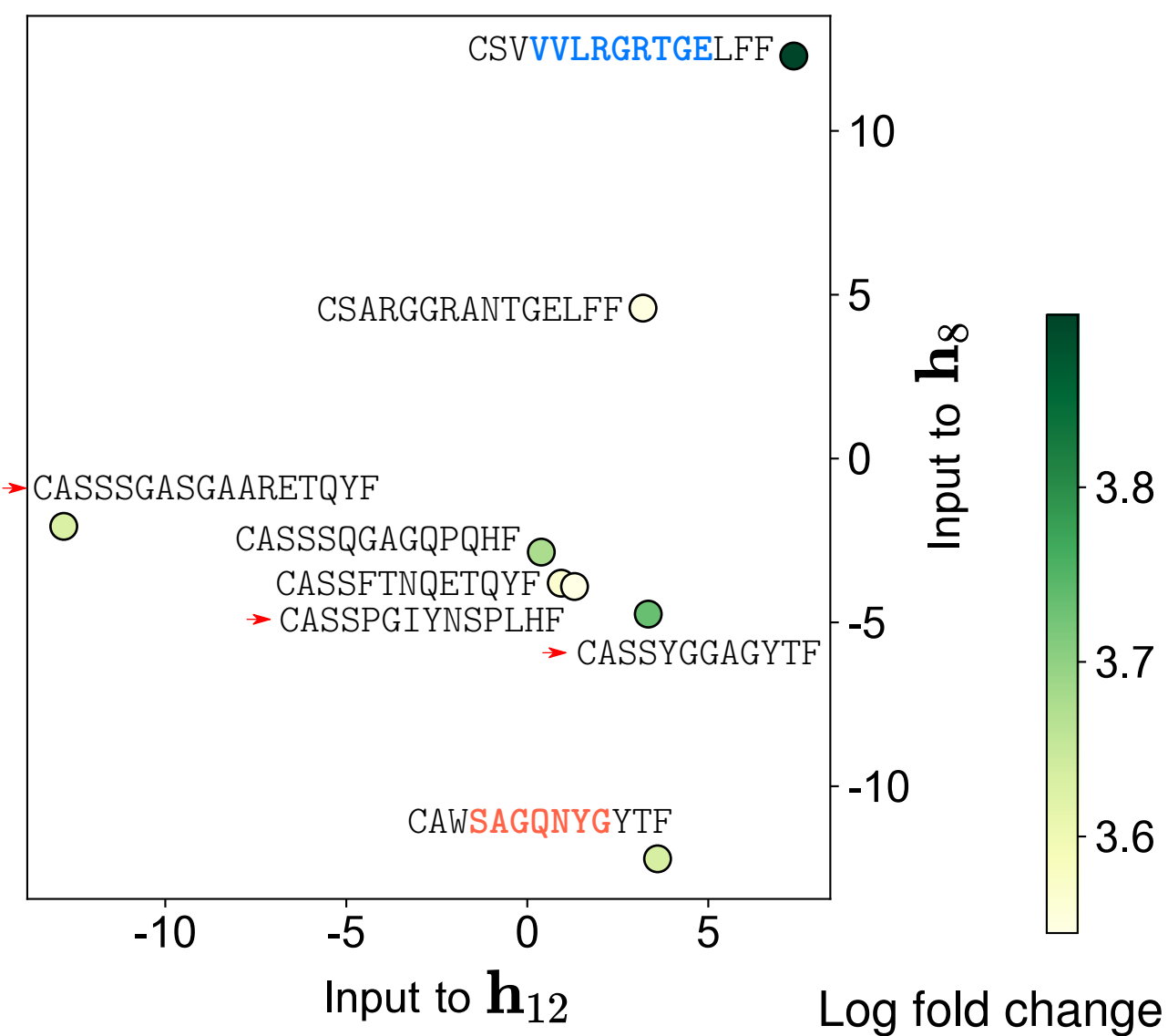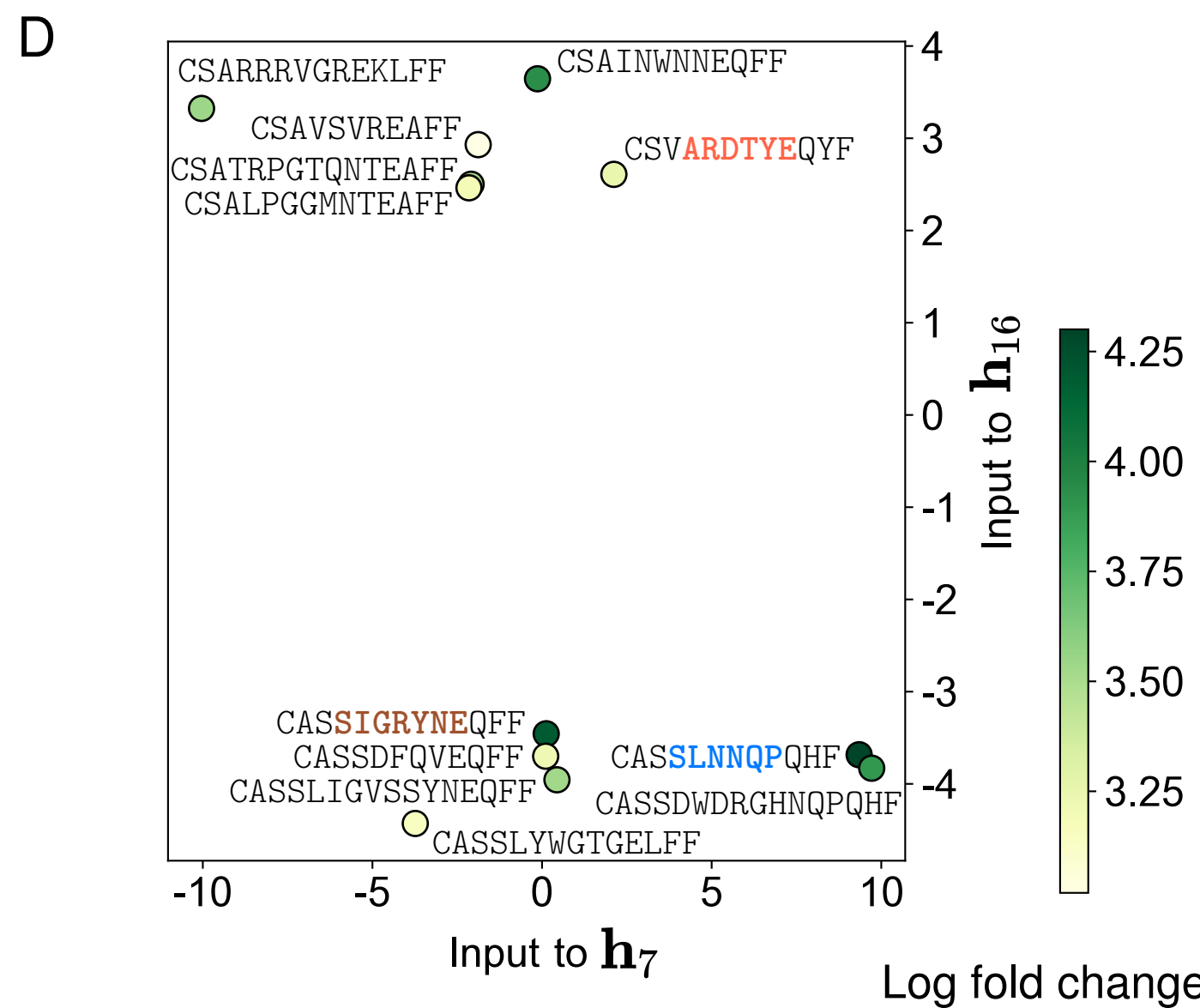

Supplement: S8 Fig — A-B: Clustering by GLIPH version 2 (GLIPH2 [23]) of the TRB sequences from respectively the datasets Pt3 NA and Pt5 CR (same datasets considered in Fig 5). We plot for each cluster the “Fisher score” reported by GLIPH2 (a p-value quantifying the significance of the motif characterizing the cluster compared to a reference repertoire) and the “expansion score” (a p-value quantifying the significance of the abundance of clones in the cluster) and we annotate the most significant clusters under both criteria by the corresponding sequence motif. GLIPH2 builds several clusters at high expansion score precisely around the motifs characteristic of expanded clones identified by RBM, as we make visible in the amino acid letters marked in color in A-B and C-D (here the same plots as in Fig 5 are reported). Clusters at highest motif-wise significance contain unexpanded clones, hence their motifs do not appear in the RBM dimensionality reduction C-D, where we kept only clones with top RBM scores (expanded). All the sequences not marked in color in C-D are expanded but not clustered by GLIPH2; in A-C, other expanded clones falling into a GLIPH2 cluster are indicated by small red arrows. We run GLIPH2 using the web tool available at http://50.255.35.37:8080/ with options: Reference version = version 2.0, Reference = CD8, all aa interchangeable = YES and providing as input for each dataset the list of CDR3 sequences, V and J segments and sequence counts at day 21. The clusters identified by GLIPH2 are 1113 for the Pt3 NA sample (total number of sequences = 14228) and 1759 for the Pt5 CR sample (total number of sequences = 14122). (PDF) [file pcbi.1009297.s008.pdf]

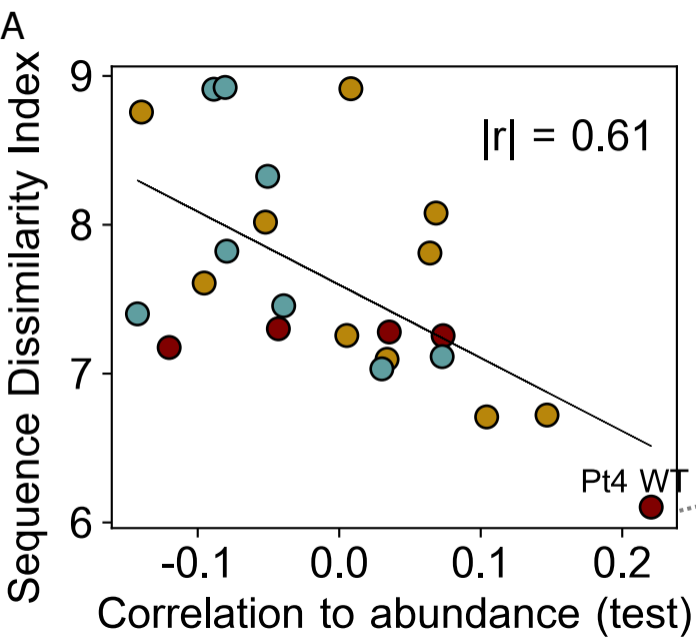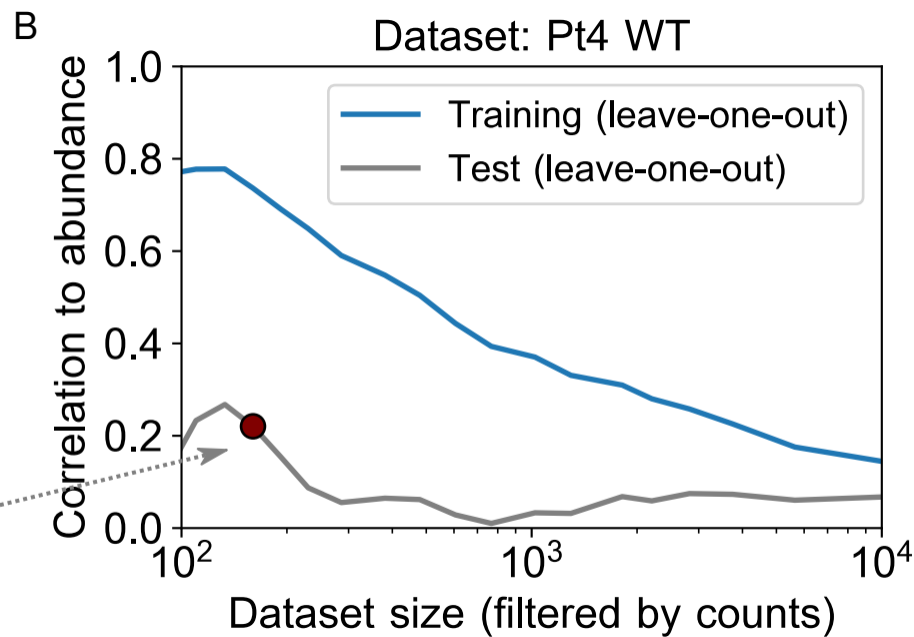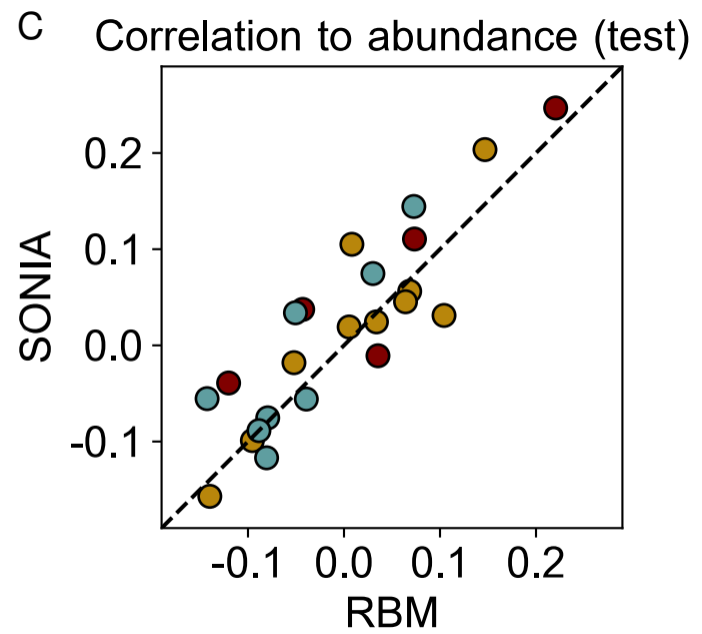

Supplement: S9 Fig — A: The x-axis gives the correlation, averaged over trainings, between the RBM score logP21p and clonal abundance 21 days post-stimulation in a 5-fold leave-one-out validation protocol (see Materials and methods) for all the Pt1,…,Pt7 datasets from [16]. The scatter plot shows that the ability of the RBM score to recover clonal abundance increases for low diversity repertoires (Pearson correlation r of magnitude |r| = 0.61, p-value for testing non-correlation = 0.002). B: Correlation between the RBM logP21p and clone abundance for the dataset at lowest dissimilarity index (Pt4 WT), both for training and testing set, as a function of the dataset size (filtered by counts). A correlation in the testing set higher than zero is recovered only when retaining the most abundant clones: the dataset size chosen for the points in A,C—indicated by the dark red dot—corresponds to ∼160 sequences. The trend, markedly different for training and testing set, signals overfitting that is unavoidable when the response is heterogeneous (as quantified by the sequence dissimilarity index, Fig 7A). C: Correlation to clone abundance by RBM—same quantity as in A—is compared to the one obtained by SONIA. (PDF) [file pcbi.1009297.s009.pdf]
