# Supplementary material for: Probing T-cell response by sequence-based probabilistic modeling
Source: PLoS Comput Biol. 2021 Sep 2;17(9):e1009297. doi: 10.1371/journal.pcbi.1009297 (PMC8476001; doi:10.1371/journal.pcbi.1009297)

RBM-LR $P_{21}^{NA}(\boldsymbol{\sigma}) \sim e^{\sum_{i=1}^{N^\sigma}(g_i^L(\sigma_i)+g^R_{i-N^\sigma-1}(\sigma_i))+\sum_{\mu=1}^{N^h}\Gamma_\mu\left(\sum_i \underbrace{(w^L_{i,\mu}(\sigma_i)+w^R_{i-N^\sigma-1,\mu}(\sigma_i))}_{\text{Weights (Left-Right)}}\right)}$

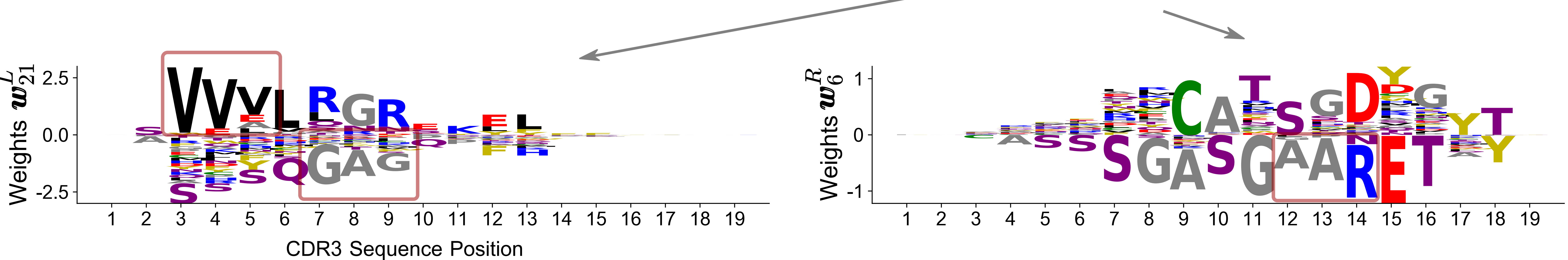

Supplement: S2 Fig — Same representation as in Fig 2A where, by starting with CDR3s in the Left+Right encoding, 2 sets of RBM weights, wL and wR, are inferred. We selected for illustration w21L and w6R as they capture the sequence motifs of responding clones highlighted in Fig 5A (dark red boxes). (PDF) [file pcbi.1009297.s002.pdf]
