# Supplementary material for: Probing T-cell response by sequence-based probabilistic modeling
Source: PLoS Comput Biol. 2021 Sep 2;17(9):e1009297. doi: 10.1371/journal.pcbi.1009297 (PMC8476001; doi:10.1371/journal.pcbi.1009297)
Supplement: Attachment — Submitted filename: revision_bravi2021.pdf [file pcbi.1009297.s013.pdf]

Revision of the manuscript ‘Probing T-cell response by sequence-based probabilistic modeling’ by Bravi et al. submitted to PLoS Computational Biology

First we thank the referee for their review and their comments which helped us improve the presentation and the discussion of our results. In the following we provide a point by point answer to the referees (in purple), all changes mentioned are marked in blue in the manuscript.

**Answer to the referees:**
**Reviewer #1:** In this work the Authors present a family of new probabilistic strategies to analyze T cell receptor (TCR) repertoire. The underlying idea is to construct probabilistic models on the base of clone abundances to extract TCR sequence Motifs. The Authors present 3 methods: Restricted Boltzmann Machines (RBM) on previously aligned TCR sequences, a selection-factor based model (SONIA), and a new RPM strategy that does not require previous alignment of the sequences. The application ground of the method consists of TRB CDR3 regions sequences of 7 patient repertoire sequences taken at different times (21 days apart).

The manuscript is scientifically sound and I find particularly interesting the fact that overall the RBM results used on the not-aligned dataset provides analogous results compared to the RBM on aligned sequences and SONIA. Moreover, the lower dimensional "projection" of the RBM on the latent variable space present sequence motives that were previously found in other works (notably Dash et al. [17], and Glanville et al [21]).

The manuscript is well written (apart from some issues that I will report below), and it is scientifically sound. Previous work in the field has been fairly taken into account in the bibliography.

In the following I will present a series of minor comments that the Author should address for the sake of clarity.

1. Datasets: I personally find the description of Balachandran et al [16] dataset in Section Results - Dataset Structure a bit too unforgiving for those computational biologists which do not have a solid background in immunology. In particular, without referring to the original paper, it was not clear to me the preliminary discussion on the neoantigen model. The first paragraph (lines 79-90) is really dense, and would benefit from a critical rewriting.

**Action:** we have provided a more detailed description of the dataset structure (see lines 70-96).

2. Presentation of the results: Although the discussion is nice and thorough I found a bit hard to figure out eventually which one of the three strategies was better suited for this kind of studies. Perhaps a final table (or another histogram) comparing the global and per patient AUROC for the 3 methods,

expanding somehow Fig.3 D would be helpful.

**Action:** we have added Figure 8, which includes a table summarizing the characteristics of the three approaches and two matrices comparing the performance of the three approaches across all samples from Balachandran et al. 2017. The performance indicators reported are the correlation of response scores to clonal fold change (from Fig. 3) and the AUROC of classification of different repertoires (from Fig. 4).

3. By construction (iterative alignment strategy) the structure of the aligned sequences have gaps in the center and letter at the extremities. Could the LR strategies be used (I do not know how) to prove or disprove this structure?

The Left+Right format, proposed in Sethna et al. 2020, is designed to exploit the knowledge of the structure of CDR3 sequence that is the result of VDJ recombination, i.e. the starting site is a cysteine encoded by the V segment, the ending sites are F/V encoded by the J segment and there is variability in sequence composition concentrated in the middle, arising from untemplated insertions and deletions. The final structure of the alignment, with gaps located in the central variable region, well reflects this known structure. We can hence say that the biological knowledge upon which the Left+right format is based proves that the alignment obtained is biologically sensible.

**Action:** we added some sentences to better explain the motivation underlying the left-right encoding and to stress the consistency of the alignment with it at lines 159-169.

**Reviewer #2:**
Bravi et al. proposes to use a machine-learning approach known as Restricted Boltzmann Machine (RBM) to identify antigen-specific TCRs with a sequence-based inference approach. Several improvements and clarifications are needed so that biologists/immunologists (and people outside the field of machine learning) could appreciate the results of the paper.

1. Figure 1 shows the schematic of the approach. I found Figure 1A and its description in the text to be confusing as it is missing some important immunological details and may be misleading. First, not only antigen but also cytokines should be added to maintain the T cells during 21 days. Second, peptides decay quickly, so T cells probably were restimulated several times between day 0 and day 21, and it should be indicated on the schematic. Third, you need to say how long the cells were stimulated at day 21 before they were taken for analysis.

**Action:** we have added the requested immunological details (addition of cytokines, the rounds of restimulation and the time of restimulation at day 21) in the schematic of Fig. 1A. We have also added these details when describing the dataset structure and the experiments (lines 97-101).

2. It looks like the patterns ("constraints") for antigen recognition are very specific for each person/antigen. Do you need the individual person/antigen data for PBMCs expansion in vitro to

obtain these patterns for a new patient or already analyzed patients and single time point data from a new patient would be sufficient? It is not clear from the description of the results how it could be generalized.

For the problem of characterizing neoantigen-specific T-cell clones, the information from already analyzed patients will in general be insufficient for new predictions since both the immunodominant neoantigens and immune response are largely individualized.
On the other hand, given a new patient, we believe that single time point data would be sufficient to infer scores of response and to extract sequence features underlying response. We can actually compare, through our probabilistic modelling approach, the single time point data with any baseline distribution describing a normal, unstimulated repertoire (such as the distribution of sequences artificially generated from models of naive repertoires). We show the robustness of our results to the choice of such baseline repertoire in Fig. 3A, where the lines indicated by 'p_gen' and 'p_post' stand for choosing the distribution of artificially generated sequences (see for explanation Fig. 3 caption and lines 209-213).

**Action:** We have stressed in the discussion that already analyzed patients carry limited information when assessing the individual response to neoantigens (lines 346-355). We have emphasized in the discussion the robustness of our predictions to the choice of the baseline distribution, supporting the applicability of our approach with single time point datasets (370-375).

For example, how much can be inferred from a single patient's blood draw (analog of PBMCs at day 0 in Figure 1A)? Will it be possible to infer the number of neoantigens (important prognostic factor) from a single blood draw? What determines the percentage of well-clustered and expanded sequences in different individuals? Please, describe the practical use of the results for the biologists/immunologists more clearly in the main text, discussion, and abstract.

By our approach, we can identify groups of TRB clones that are expanded and that are clustered as they share particular sequence motifs. One can hypothesize that different clustered groups of TRBs correspond to responses to different targets but, without additional experimental tests on the reactivity of single TCRs, we cannot draw conclusions about the number of targeted antigens. In particular, we found that well-defined clusters clearly corresponded to different viral epitopes the case for tetramer-sorted data while in the neoantigen stimulated data we observed a lower degree of repertoire focusing around one or a few well-defined clusters, as we discuss at line 409-411, hence also the type of assay will affect the extent to which we can infer the number of targets from the degree of clustering.

Our method can give a measure of the degree of specificity of response in a sample and identify sequence features that are shared among responding clones. Identifying the factors determining the percentage of well-clustered and expanded sequences in different individuals is beyond the predictive power of our method, since it would require better knowledge of the actual TCR-peptide binding modes and, in cancer, of the tumor's characteristics that are susceptible to produce viable neoantigens in large cohorts of patients. For the particular case of pancreatic

cancer, it is difficult to collect a large number of datasets describing the tumour mutation profile (giving rise to neoantigens) and the T-cell response due to the rarity of long-term survivors. With more data of this type becoming available, we would be interested in correlating antigen biophysical properties to the degree of clustering in the TCR response.

**Action:** we have added some sentences along the lines of these answers in the discussion section, both when we stress the limitations of our approach and when we mention the future directions we are interested in (378-388).

3. Please describe the limitations: what the method could and could not do in terms of answering the related biological questions.

**Action:** we have added a long paragraph of discussion on the limitations of our approach (lines 378-396), incorporating the points discussed in the previous answers. We have also expanded the discussion of the specificity measure we propose (lines 233-240) to highlight its potential use to identify immunostimulant peptides.

4. The last sentence in the abstract is unclear. I suggest replacing it with a more specific example(s) of practical use of the RBM approach (see point 2).

**Action:** we have modified the last sentence of the abstract adding a precision on the type of datasets and experiments to which our approach is aimed.

Regarding data and code availability, all the dataset and codes used to produce the results are now publicly available at https://github.com/bravib/rbm_tcell, as we indicate in the ‘Data Availability Statement’. Tables with the numerical values used for Figs. 3,4,7,8 are provided as Supporting Information. The visualization of data and models’ parameters of Figs. 1,2,5,6 is illustrated in the python notebooks contained in the Git repository.

In terms of references, we have added the following ones:

- Ref. 20, a reference for the process of VDJ recombination;
- Ref. 31, to provide a review on the neoantigen discovery problem.

Finally, we have made other minor, formal changes not required by the referees (always marked in blue) that concern:

- Mathematical notation: to stick to the same notation used by the some of the authors in other papers (see e.g. Sethna et al 2018, Sethna et al 2020), we changed the notation of p_gen and p_post, in particular we now use capital P and roman font for the subscript.

For consistency, we now use capital P everywhere to indicate a probability and roman font for all subscripts.

- The name ‘Diversity Index’: we have realized that this metric, based on the amino acid sequence dissimilarity, could be confused for other metrics of diversity of immune repertoires that are rather more focussed on clone frequency distribution. To better stress the role of sequence dissimilarity, we switched from ‘Diversity Index’ to ‘sequence dissimilarity index’ (see for example Fig. 7 and S9).

We have also added a small inset in Figure S3 to better clarify the sentence at lines 193-194.
